# Supplementary material for: Comprehensive Annotation and Functional Exploration of MicroRNAs in Lettuce
Source: Front Plant Sci. 2021 Dec 24;12:781836. doi: 10.3389/fpls.2021.781836 (PMC8739914; doi:10.3389/fpls.2021.781836)
Supplement: Supplementary file 3 [file Data_Sheet_9.ZIP › GSM2230752.plot/Lsa-miR6114_Lsat_1_v5_gn_5_44701.1_5218_TPlot.pdf]

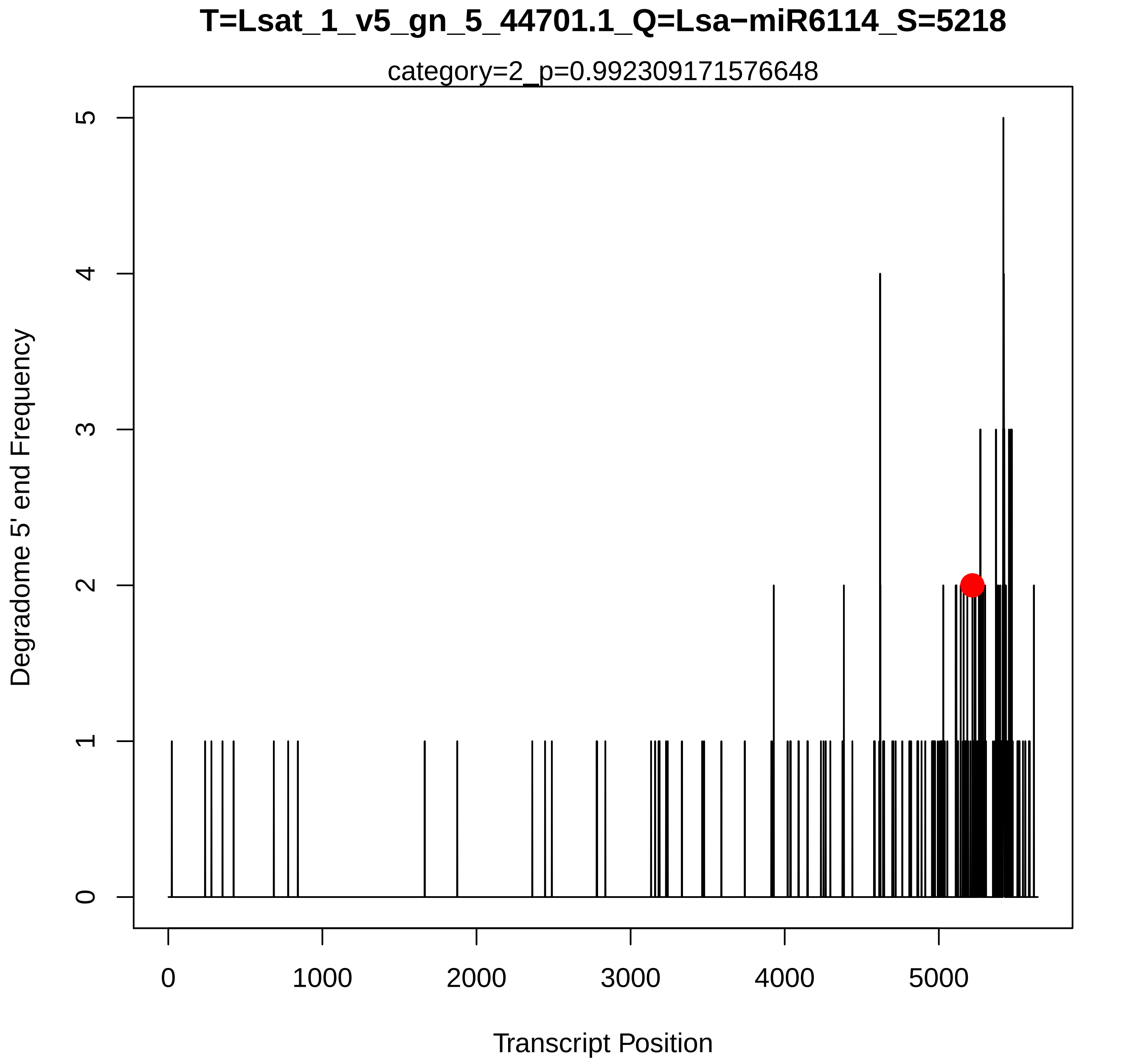

T=Lsat_1_v5_gn_5_44701.1_Q=Lsa−miR6114_S=5218
category=2_p=0.992309171576648
Degradome 5' end Frequency
0
1
2
3
4
5
0
1000
2000
3000
4000
5000
Transcript Position
